# A rare mediastinal mass treated by submucosal tunneling endoscopic resection

**DOI:** 10.1055/a-2561-1303

**Published:** 2025-03-28

**Authors:** Weizhao Wang, Mengxian Ju, Jun Liu, Pinghong Zhou, Lu Wang, Chaowu Chen, Chao Sun

**Affiliations:** 1 370089 Endoscopy Center, Northern Jiangsu Peopleʼs Hospital Affiliated to Yangzhou University, Yangzhou, China; 2 370089 Endocrinology, Northern Jiangsu Peopleʼs Hospital Affiliated to Yangzhou University, Yangzhou, China; 3 92323 Endoscopy Center, Zhongshan Hospital Fudan University, Shanghai, China

A 46-year-old man presented with a sensation of obstruction during eating and gastroscopy revealed a submucosal mass in the lower esophagus. The mass was located about 35–38 cm from the incisors, had a smooth surface, and measured 3.0 × 5.0 cm ( Fig. 1 ). Endoscopic ultrasound examination revealed a hypoechoic mass protruding into the esophageal and mediastinal cavities, which was poorly demarcated from the muscularis propria, and was considered to have originated from either the muscularis propria or the mediastinum as a space-occupying lesion ( Fig. 2 ). A enhanced computed tomography scan showed a hypodense mass of homogeneous density adjacent to the lower esophagus, about 3.0 cm in diameter, poorly demarcated from the esophagus and mediastinum, with unremarkable enhancement ( Fig. 3 ). The patient’s full blood count, liver, renal, and coagulation function, and tumor markers were within reference values. The preoperative diagnosis was of “submucosal tumor of the esophagus” or “mediastinal occupation.” Endoscopic resection (submucosal tunneling endoscopic resection [STER]) was performed after obtaining consent from the patient.

**Fig. 1:**
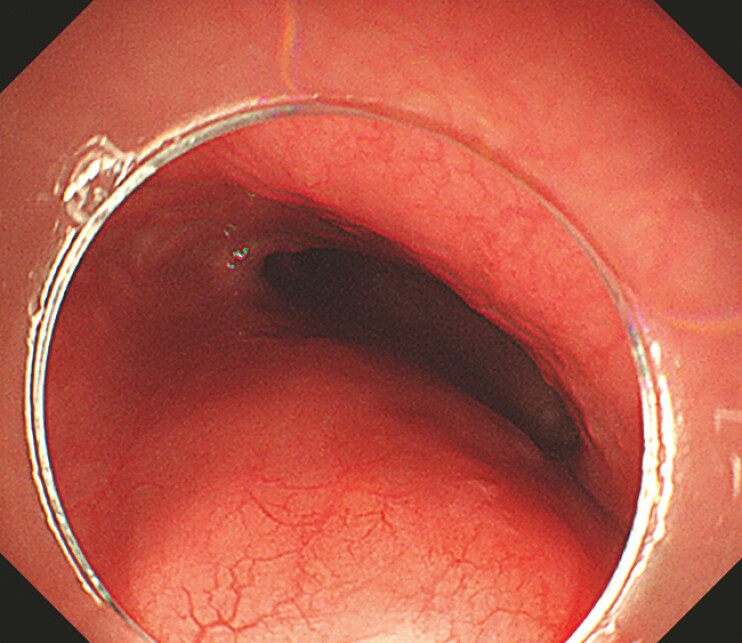
Gastroscopic image showing a mass with a smooth surface in the esophageal wall, located 35–38 cm from the incisors.

**Fig. 2:**
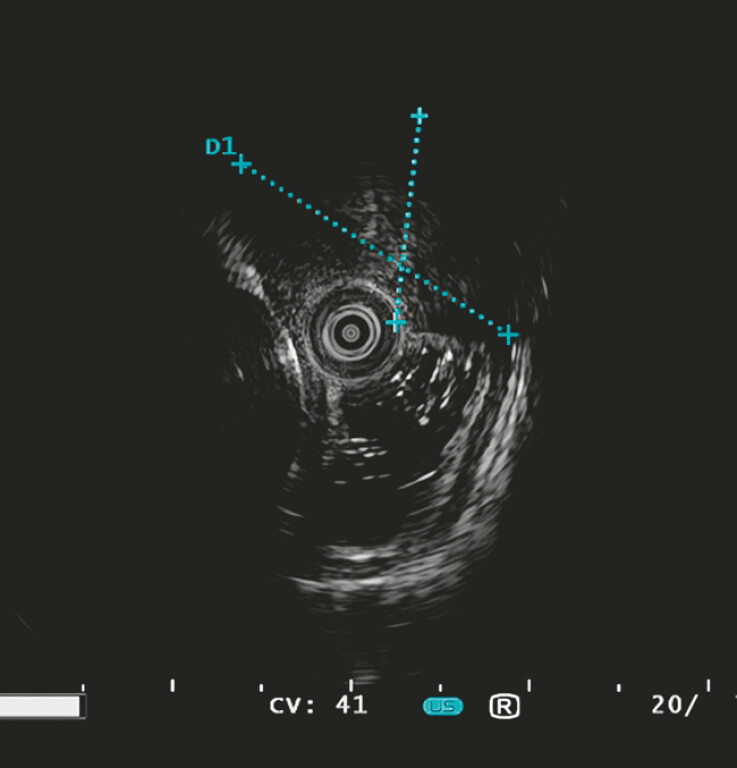
Endoscopic ultrasound image showing a hypoechoic mass protruding into the esophageal and mediastinal cavities, which was poorly demarcated from the muscularis propria.

**Fig. 3:**
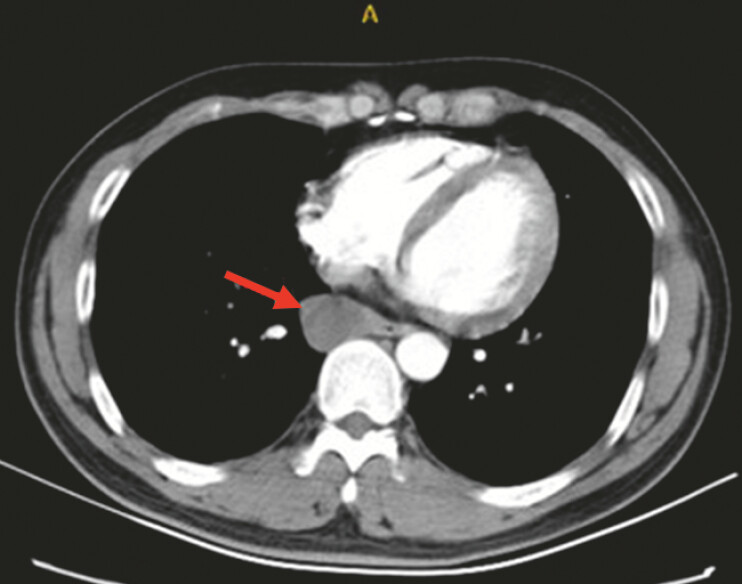
Enhanced computed tomography scan showing a hypodense mass of homogeneous density adjacent to the lower esophagus, about 3.0 cm in diameter, poorly demarcated from the esophagus and mediastinum, with unremarkable enhancement.

After establishing a submucosal tunnel to reach the tumor site, we found that the mass was located at a deeper level. Therefore, we incised the muscularis propria and the outer esophagus into the mediastinum to completely remove the mass. Intraoperatively, mucus and jelly-like fluid were seen oozing from the mass. The tumor was successfully removed under gastroscopic guidance ( Video 1 ), with the resected specimen measuring 4.0 × 5.0 cm ( Fig. 4 ). Histopathologic examination revealed the tumor to be a cyst with a wall composed of fibrous tissue lined with ciliated columnar epithelium, and a mediastinal cyst was diagnosed ( Fig. 5 ). The patient remained asymptomatic over 12 months of follow-up.

A mediastinal cystic mass is successfully resected using the submucosal tunneling endoscopic resection procedure, with only minor bleeding experienced during the procedure.Video 1

**Fig. 4:**
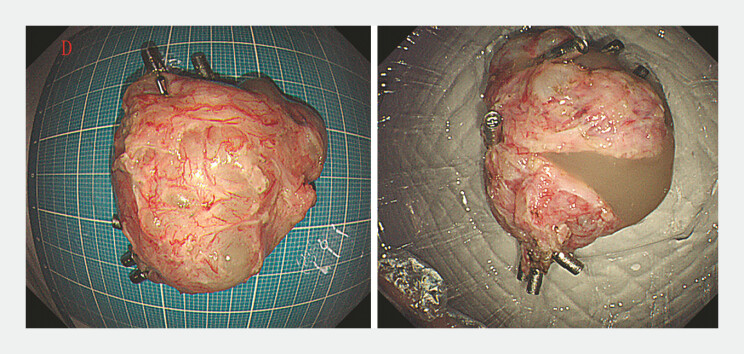
Photographs of the resected specimen, which measured 4.0 × 5.0 cm and was a cystic mass containing a large amount of mucus and jelly-like fluid.

**Fig. 5:**
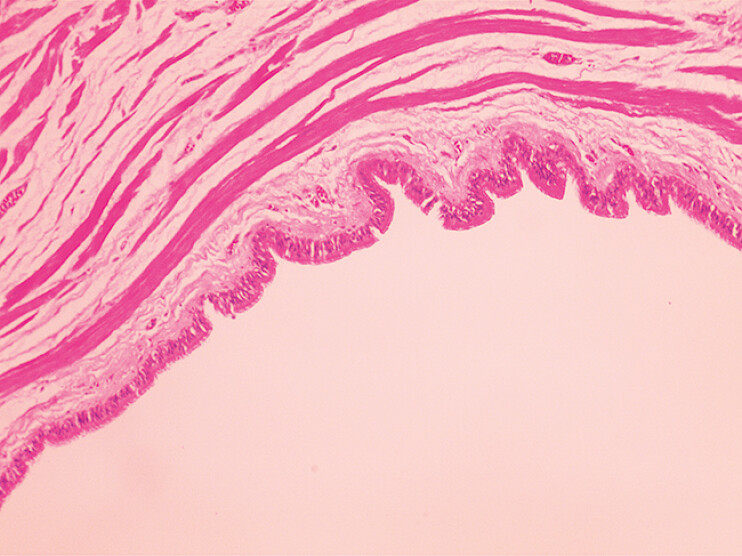
Histopathologic appearance showing that the tumor was a cyst, with its wall composed of fibrous tissue lined with ciliated columnar epithelium.

Mediastinal cysts are cystic benign lesions that occur in the mediastinum, grow slowly, rarely become malignant, and are treated mainly by surgery 1 2 3 . To the best of our knowledge, this if the first report of a mediastinal cyst being treated by gastroscopy in the English literature. This case reminds endoscopists that mediastinal cysts can protrude into the lumen of the esophagus and may be misdiagnosed as submucosal tumors of the esophagus. Although imaging and ultrasound can provide some information, the final diagnosis usually depends on histopathologic examination of the resected specimen, and our approach provides a new idea for minimally invasive treatment of mediastinal cysts.

Endoscopy_UCTN_Code_TTT_1AO_2AG_3AZ

## Notes

Endoscopy E-Videos https://eref.thieme.de/e-videos
*E-Videos* is an open access online section of the journal *Endoscopy* , reporting on interesting cases and new techniques in gastroenterological endoscopy. All papers include a high-quality video and are published with a Creative Commons CC-BY license. Endoscopy E-Videos qualify for HINARI discounts and waivers and eligibility is automatically checked during the submission process. We grant 100% waivers to articles whose corresponding authors are based in Group A countries and 50% waivers to those who are based in Group B countries as classified by Research4Life (see: https://www.research4life.org/access/eligibility/ ). This section has its own submission website at https://mc.manuscriptcentral.com/e-videos .
